# Vasorelaxant Effects of the *Vitex Agnus-Castus* Extract

**DOI:** 10.1155/2022/7708781

**Published:** 2022-03-22

**Authors:** Shpëtim Thaçi, Berat Krasniqi, Miribane Dërmaku-Sopjani, Arleta Rifati-Nixha, Sokol Abazi, Mentor Sopjani

**Affiliations:** ^1^Faculty of Medicine, University of Prishtina, Prishtina 10000, Kosovo; ^2^Faculty of Natural Sciences and Mathematics, University of Prishtina, Prishtina 10000, Kosovo; ^3^Canadian Institute of Technology, Tirana 100, Albania

## Abstract

This study was undertaken to describe and characterize the relaxing effects of the medicinal plant *Vitex agnus-castus* (VAC) extract on isolated rabbit arterial rings. The VAC extracts (VACE) were extracted with ethanol and tested in aorta rings (3-4 mm) of rabbits suspended in an organ bath (Krebs, 37°C, 95% O_2_/5% CO_2_) under a resting tension of 1 g to record isometric contractions. After the stabilization period (1-2 hours), contractions were induced by the addition of phenylephrine (0.5 *μ*M) or high KCl (80 mM) and VACE was added on the plateau of the contractions. Experiments were performed to determine the effects and to get insights into the potential mechanism involved in VACE-induced relaxations. The cumulative addition of VACE (0.15–0.75 mg/mL) relaxed, in a concentration-dependent manner, the rabbit aorta rings precontracted either with phenylephrine- or with high KCl thus suggesting calcium channel blocking activities. The VACE effect appeared to be endothelium-dependent. The preincubation with L-NAME (the inhibitor of nitric oxide synthases (NOS)), ODQ (the selective inhibitor of guanylyl cyclase), and indomethacin (the cyclooxygenase inhibitor), downregulated VACE-induced relaxation of aorta rings precontracted with phenylephrine, whereas the bradykinin (stimulator of NOS) and zaprinast (phosphodiesterase inhibitor) further upregulated relaxant effects induced by VACE. These results revealed that the aorta relaxation effect of VACE was mainly endothelium-dependent and mediated by NO/cGMP and prostaglandins synthesis. This vasodilator effect of VACE may be useful to treat cardiovascular disorders, including hypertensive diseases.

## 1. Introduction

Herbal medicine has been commonly used throughout human history for the treatment of various conditions, or diseases [1]. Medicinal herbs have been widely used for both culinary and medicinal purposes and played an important role in the development of human culture. Herbal medicine-derived natural products have been used in traditional medicine for the prevention and treatment of various human conditions, including cardiovascular diseases [2, 3].

Numerous plant-derived natural products have already been isolated [1, 4–7], with a great potential to be used for healing purposes in a variety of disorders [7–11]; many of them have already been approved for therapeutic use in the last years [12, 13]. However, the effects of some of them still need to be mechanistically characterized. Therefore, pharmacological characterization of their effects, and especially the underlying intracellular mechanism involved, may help to better understand their roles and can be used as a strategy for identifying new applicable and more potent drugs.

The genus *Vitex* L. (family *Lamiaceae*) has about 250 different species. These are deciduous shrubs, mainly native throughout the tropic and subtropics regions. One of the *Vitex* species is *Vitex agnus-castus* (chaste tree, VAC) [14, 15]. It is an aromatic, ornamental, and deciduous shrub native to the Mediterranean and Western Asia. The chemical composition of VACE (*Vitex agnus-castus* extract) was reported [16] in 2016, revealing the presence of 47 different components accounting for about 99% essential oils from the leaves, fruit, and inflorescence, respectively. Lately, our team published the tracheorelaxant properties and mode of action of VACE fruits, as well as their phytochemical composition [17]. Fruits of VACE have been used for the treatment of many female conditions, including hormonal imbalances, pain, and menstrual cycle problems [14, 15, 18–20]. Noteworthily, in Albanian folk medicine, VAC fruits and leaves are often used for increasing milk and female reproductive disorders treatment.

Additionally, the extracts from genus *Vitex* have been reported to have cardiovascular activities [15] and were used to treat hypertension. However, these functions are mainly not scientifically assessed. Recently, the leaf extracts of *Vitex pubescens* have been reported to have antihypertensive and vasorelaxant action in the aorta [21]. Therefore, this study was aimed to investigate the vascular effects of *Vitex agnus-castus* extract on rabbit aorta rings under various experimental conditions.

## 2. Materials and Methods

### 2.1. Preparation of the Crude Plant Extract

The fruits of VAC (*Vitex agnus-castus*, L) were collected carefully from Albania. Plant materials were professionally determined by a botanist and then manually picked, cleaned up of adulterants, and shade-dried away from strong winds [17, 22]. Then, it was further grinded to a coarse powder by a grinding machine. The resulting material was soaked in 80% ethanol for 24 hours at room temperature under constant shaking and then filtered by passing through a filter paper. After that, the filtered liquid (filtrate) was concentrated in a rotary evaporator under pressure, dried out, and transferred to containers. The extract was standardized with 0.13–0.15% casticin and kept in a refrigerator (4°C). Appropriate dilutions of the crude plant extract from the stock were freshly made on the day of the experiment.

### 2.2. Reagents

Phenylephrine (PE, a vasoconstrictor), N^G^-nitro-L-arginine methyl ester (L-NAME, an inhibitor of nitric oxide synthases (NOS)), bradykinin (a stimulator of NOS), indomethacin (a cyclooxygenase (COX) inhibitor), ODQ (1H- (1,2,4) oxadiazolo (4,3-a) quinoxalin-1-one, a selective inhibitor of soluble guanylyl cyclase (sGC)), and zaprinast (a selective inhibitor of cGMP-specific phosphodiesterases V and VI (PDE5/6)) were purchased from Sigma Aldrich, Germany. All chemicals and reagents used for making physiological salt solutions and other analyses were of analytical grade. Bradykinin was dissolved in 0.1 M acetic acid; indomethacin was dissolved in ethanol, while zaprinast was dissolved in DMSO at 10 mM. Unless otherwise specified, all the drugs were dissolved in distilled water [17, 22]. Of note, all experimental solutions of drugs were made fresh daily.

### 2.3. Treatment and Sensitization of Rabbits

Adult rabbits (standard chinchilla rabbits), local breed and either sex, weighing about 800–1200 g (Gram), were treated according to the law of animals' protection of the Republic of Kosova (ethics committee approval no. AUV-03; 1557). The experiments were performed according to the national and international standards for animal research [23, 24], in compliance with the European Council Directive of November 24, 1986 (86/609/EEC). Rabbits were kept in proper conditions, 19–23°C, 12 hours light/dark regimen cycle, and given ad libitum food and water. Animals were housed in the animal facility of the Faculty of Medicine of the Uni. of Prishtina. Rabbits of either sex were sacrificed following a blow on the back of the head and their thoracic aorta was taken out by dissection and kept in the normal Krebs–Henseleit solution (KHS) in the following composition (mM): NaCl (118), KCl (4.7), CaCl_2_ (2.52), MgSO_4_ (1.64), KH_2_PO_4_ (1.18), NaHCO_3_ (7), and glucose (5.5). Aorta was then cut vertically in 2-3 mm width rings. Each isolated aorta ring was then mounted between two stainless-steel hooks in the thermostatically controlled (37°C) organ baths. The lower hook was fixed at the bottom of the organ bath, while the upper one was connected to an isometric transducer (DMT 750, Danish Myo Technology, Denmark) connected to an ink writing recorder. The mounted aorta tissue was kept in the organ bath (10 ml) containing KHS (pH 7.4) and continuously aerated with 5% CO_2_ and 95% O_2_.

### 2.4. Experimental Protocols

Changes in isometric tension of aortic rings were continuously measured with a force transducer (DMT 750, Danish Myo Technology, Denmark). An optimal preload of 1 g was applied to each aorta ring and allowed to equilibrate for about 1 h, during which the preparations were regularly washed out with KHS every 15 min and resting tension of 1 g was readjusted. After equilibration, rings were stimulated with PE (0.5 *μ*M) or K+ (80 mM) until a sustained response was obtained. Then, VACE was added on the plateau of either PE- or K+-induced contraction in a cumulative manner (VACE 0.15 to 0.75 mg/mL) or in a single maximal concentration (VACE 0.75 mg/mL). Control preparations were treated with a drug vehicle only.

Endothelium-derived relaxing factors are known to induce aorta smooth muscle (ASM) relaxation [25]. Accordingly, we hypothesized the involvement of endothelium in the relaxant effects of VACE. To this end, in a series of experiments with aorta rings, the endothelium was removed by gently rubbing the intimal surface of the vessel with a smooth wooden stick in an appropriate condition. After preparation, successful removal of the endothelium was assessed by the significant decrease of acetylcholine (ACh, 1 *μ*M) ability to elicit the aortic ring relaxation, while smooth muscle integrity was tested with KCl (80 mM)-induced contraction in both, endothelium-intact and endothelium-denuded aorta rings. The relaxant effect of VACE was tested as explained above. To deeper investigate the endothelium-dependent mechanisms of VACE relaxation [5, 25], the endothelium-intact aortic rings were treated with PE, in presence or absence of 0.75 mg/mL of VACE, and without (PE + VACE alone) or with bradykinin, L-NAME, ODQ, zaprinast, or indomethacin, respectively [26, 27]. All abovementioned inhibitors and the stimulator were added 5 min prior to induce contraction by PE. The aortic rings evoked a constriction effect that reached the plateau level for about 30 minutes after PE treatment. The tested aortic rings were performed in parallel without or with the specific inhibitor, stimulator, respectively.

### 2.5. Statistical Analysis

The relaxant function of VACE in the aorta is expressed as a value of PE-KCl-induced maximal contractions compared to 1 g of contraction force. The data are representative of at least four independent experiments (*n* = 4) for each series of experiments and expressed as means ± SEM. Statistical analysis was made by one-way ANOVA and Dunnett's post-test. A value of *p* < 0.05 was considered statistically significant (GraphPad Prism Software, La Jolla, CA).

## 3. Results

VACE significantly relaxed the PE, -high K^+^, -precontracted aortic rings in a dose-dependent manner.

**Figure 1:**
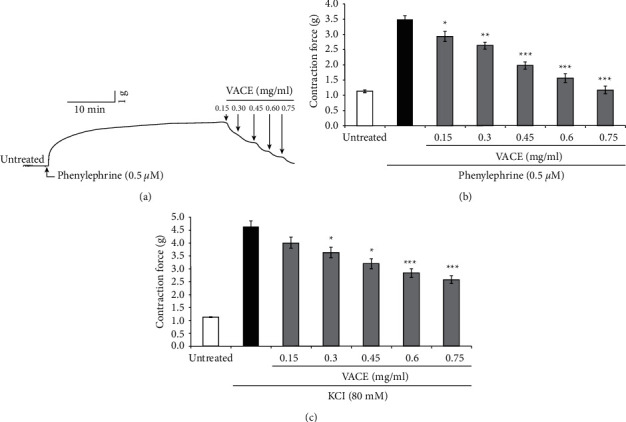
Concentration-dependent relaxant effect of VACE on PE-, KCl, -induced ASM contractions. (a). Original representative tracings in force/time of isolated rabbit ASM before (untreated) and after PE (0.5 *μ*M)-induced contraction followed by the addition of cumulative concentrations (0.15–0.75 mg/mL) of VACE. Arithmetic means ± SEM (*n* = 4-5 aortic rings, each from a different animal) of the relaxant effects of VACE cumulative concentrations (0.15–0.75 mg/mL, gray bars) on rabbit ASM induced by PE (0.5 *μ*M, black bar) (b) and KCl (80 mM, black bar), respectively (c). All results are shown as a value of the 1 g of contraction response. Statistically significant difference from the respective VACE concentration in rabbit aorta compared to PE and KCl alone, respectively.  ^*∗*^*p* < 0.05,  ^*∗∗*^*p* < 0.01, and  ^*∗∗∗*^*p* < 0.001.

In the first series of experiments, the effect of increasing concentrations of VACE on the basal tone of isolated rabbit aortic rings was assessed. As shown in Figures 1(a), 1(b), concentrations of VACE higher than 0.15 mg/mL significantly relaxed the PE-induced contraction.

In the next series of experiments, we tested the effects of cumulative concentration of VACE on high (80 mM) K^+^-induced contraction of rabbit aortic rings. As reported in Figure 1(c), concentration of VACE higher than 0.3 mg/mL significantly reduced the high K^+^-evoked aortic contraction.

### 3.1. Vasorelaxant Effects of VACE Are Partially Endothelium-Dependent

**Figure 2:**
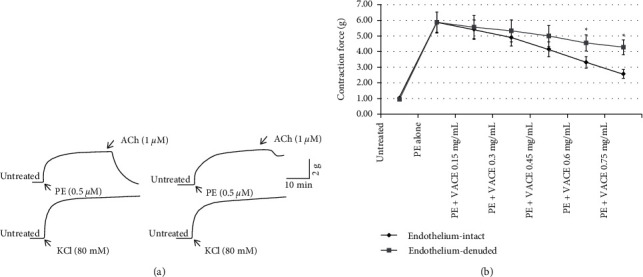
Effect of endothelial denudation on VACE relaxant effect after the PE-induced muscle contraction in isolated rabbit aorta. (a). Original representative tracings in force/time of isolated rabbit ASM before (untreated) and after PE (0.5 *μ*M)-induced, and after reaching the plateau, following the ACh (1 *μ*M)-induced relaxation to determine endothelium integrity in endothelium-intact ((a) upper left) and endothelium-denuded ((a) upper right). To test the integrity of smooth muscle after the endothelium removal, the aorta rings were precontracted with KCl (80 mM) integrity in endothelium-intact ((a) lower left) and endothelium-denuded ((a) lower right). (b) Data are shown as the arithmetic means ± SEM (*n* = 7-8) and expressed as the contraction force (g) of maximal contraction induced by PE (0.5 *µ*M) followed with VACE treatment (0.15–0.75 mg/mL) of aortic rings with or without endothelium, respectively. *n* indicates the number of different rabbits from which descending thoracic aortic rings were derived.  ^*∗*^*p* < 0.05 compared between endothelium-denuded and endothelium-intact aortic rings.

The presence or absence of an intact endothelium in all preparations was assessed by testing the capacity of ACh to induce relaxation of rings precontracted with phenylephrine.  Figure 2(a) shows almost complete loss of relaxing response of aorta rings to ACh on endothelium-denuded as compared to endothelium-intact preparations, which were used to test the role of the endothelium in VACE-induced ASM relaxation. The steady contraction of rabbit aortic rings precontracted with PE was similar in the endothelium-intact and endothelium-denuded rings. The addition of VACE (0.15–0.75 mg/mL) induced a concentration-dependent relaxation, which was significantly higher in endothelium-intact than in endothelium-denuded aortic rings (Figure 2(b)). Thus, the vasorelaxant effect of VACE appears to be at least partly endothelium-dependent.

### 3.2. Involvement of NO/cGMP Signaling Pathway in VACE-Induced Vasorelaxant Activity

**Figure 3:**
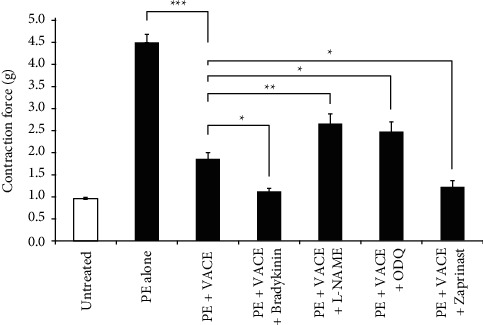
Bradykinin and zaprinast upregulated, whereas L-NAME and ODQ downregulated the relaxant effects of VACE in aorta rings precontracted with PE. Arithmetic means ± SEM (*n* = 5–12) of the basal tonus (1-st bar) and the relaxation responses precontracted with PE (0.5 *µ*M) alone (2-nd bar) PE and (0.75 mg/mL) VACE (3-rd bar) PE and (100 nM) bradykinin and VACE (4-rth bar, PE and (100 *µ*M) L-NAME and VACE (5-th bar) PE and (10 *µ*M) ODQ and VACE (6-th bar), or PE and (10 *µ*M) zaprinast and VACE (7-th bar). In all cases where indicated, bradykinin, L-NAME, ODQ, and zaprinast were preincubated for 5 min. Statistical significance ( ^*∗*^*p* < 0.05,  ^*∗∗*^*p* < 0.01, and  ^*∗∗∗*^*p* < 0.001) between treatments, was as indicated in the figure. VACE-induced aortic relaxation involves prostaglandins production.

To further explore the endothelium participation in VACE-induced vasodilatation, we tested the involvement of the nitric oxide (NO)-cyclic GMP (cGMP) pathway. Therefore, as presented in Figure 3, the relaxant effect of VACE (0.75 mg/mL) on PE-induced arterial contraction was significantly increased by tissue incubation with bradykinin (nitric oxide synthase stimulator, 100 nM) (*p* < 0.05, *n* = 9) or zaprinast a selective inhibitor of cGMP-specific phosphodiesterases V and VI (PDE5/6, 10 *µ*M) (*p* < 0.05, *n* = 9), whereas L-NAME (a nonspecific nitric oxide (NO) synthase inhibitor, 100 *μ*M) (*p* < 0.05, *n* = 10) or ODQ (a selective inhibitor of sGC, 10 *μ*M) (*p* < 0.05, *n* = 5), significantly decreased the VACE-induced dilatation (*p* < 0.001, *n* = 12) of isolated rabbit aortic rings as compared to VACE following PE-induced aortic precontraction alone.

**Figure 4:**
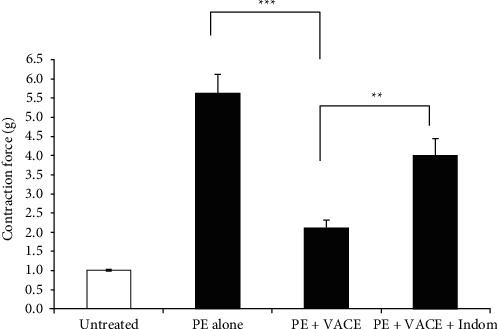
Indomethacin inhibited the VACE-induced relaxant effects of rabbit aortic rings precontracted with PE. Arithmetic means ± SEM (*n* = 4–8 aortic rings, each from a different rabbit) of the measurements recorded in rabbit ASM with PE (0.5 *µ*M) and treated with VACE 0.75 mg/mL without (PE + VACE) or with (after 5 min pretreatment) 10 *µ*M indomethacin (PE + VACE + Indomethacin). Significant differences from the absence of VACE, as well as from the absence of indomethacin:  ^*∗∗*^*p* < 0.01,  ^*∗∗∗*^*p* < 0.001.

Another mechanism of vascular relaxation involves prostaglandins (PG) production [28, 29], mediated by COX induction [30]. To determine whether PG was also involved in VACE-induced relaxation of rabbit aorta, the effect of the extract was evaluated in PE-contracted rings preincubated with indomethacin (10 *μ*M). The nonselective cyclooxygenase inhibitor significantly reduced the vasodilatory response of the vessels to VACE (Figure 4) (*p* < 0.01, *n* = 4–8).

## 4. Discussion

This study showed for the first time that the VACE has vasorelaxant effects on rabbit aortic rings contracted by either PE or high K^+^ (Figures 1(a), 1(b) and 1(c)), which indicated that the *Vitex agnus-castus* contains bioactive compounds capable of regulating the function of blood vessels. Extracts or specific isolated compounds of genus *Vitex* have been previously shown to exert numerous functions and used for treating certain menstrual disorders, infertility, hyperprolactinemia, acne, corpus luteum insufficiency, PMDD, menopause, cyclical mastalgia, inflammatory conditions, and cyclic breast pain, disrupted lactation, diarrhea, and flatulence [14, 15, 18–20]. In Albanian folk medicine, fruits and leaves of VAC are frequently used for the treatment of numerous female reproductive problems. The phytochemical composition of VACE has been previously reported [16], including a paper from our team on their tracheorelaxant properties and underlying mechanisms [15]. The extracts from other species (*Vitex pubescens*) of genus *Vitex* have been reported to have cardiovascular activities [15]. However, to our best knowledge, the vascular relaxant effects and underlying mechanisms of *Vitex agnus-castus* described in the present work have not been reported earlier. The effect of VACE was observed at a relatively narrow range of concentrations.

VACE-mediated vasorelaxant effects may be both endothelium-dependent and endothelium-independent. The present study showed that PE and high K+-induced contraction of aortic smooth muscle is associated with increased intracellular calcium levels [5, 31, 32]. Calcium can regulate either the production or release of various endothelial-derived relaxing factors [33]. Nonspecific inhibition of both PE and high K+ by VACE suggests that its nonspecific vasorelaxant role, at least partly, may involve calcium channel blockade mechanisms. These results are consistent with our previous study reporting a tracheorelaxant function of VACE [17]. This VACE relaxant function may partly be endothelium-independent, potentially through the inhibition of calcium receptors, either extracellular and/or intracellular, and by activation of K_ATP_ channels [21]. This does not exclude the involvement of other potential mechanisms, such as inhibition of *β*-adrenergic receptors in ASM cells [33]. Calcium channel blocking properties of VACE in ASM may have antioxidant functions [7, 15].

Endothelial cells are essential in the control and regulation of various physiological cardiovascular functions [34]. Endothelium deregulation is associated with diseases, including cardiovascular diseases [35]. In this regard, the endothelial cells appear to be the main site of the relaxant action of the bioactive compounds present in the VACE. Although the effect of VACE was observed in both preparations; however, VACE-induced relaxation at higher concentrations-in denuded aortic rings precontracted by PE was significantly lower than intact aortic rings (Figure 2). The presence of the VACE effect in both types of preparations might be due to either endothelium was not fully absent in endothelium-denuded preparations or the involvement of endothelium-independent mechanisms in VACE effects. Herein, it may be that VACE could be acting on the ASM cells to increase responsiveness to endothelium relaxing factors [36]. The VACE-induced endothelium-mediated ASM relaxation effects seem to be particularly associated with a synergic induction of NO/cGMP and PG signaling pathways. This particular VACE endothelium-dependent activity suggested a potential role for NO release or activation and the cyclooxygenase signaling pathway and/or their interactions on vascular smooth muscles [37].

Accordingly, we found the effect of L-NAME, ODQ, bradykinin, and zaprinast (Figure 3), as well as indomethacin on VACE relaxation (Figure 4). In line with this, one of the well-known mechanisms that induce vascular smooth muscle cell relaxation, including vascular smooth muscle cells, is the NO/cGMP pathway [37, 38]. Nitric oxide is a ubiquitous molecule, an endogenous vasorelaxant mediator that under normal conditions or in response to a variety of agonists can be generated by the NOS isoforms, which use L-arginine, oxygen, and NADPH as substrates to generate NO and L-citrulline [39]. sGC is the receptor for NO in vascular smooth muscles. NO activates sGC, which in turn catalyzes the conversion of guanosine triphosphate (GTP) to the intracellular second messenger cyclic guanosine monophosphate (cGMP) in vascular smooth muscle cells, and thereby increased levels of cGMP induces muscle relaxation [37, 39, 40]. On the contrary, PDE5-catalyzed cGMP degradation to GMP accounts for ending vasorelaxation [25, 40]. As a deduction, PDE5 inhibitors, such as zaprinast, inhibit the degradation of cGMP leading to induced muscle relaxation. Accordingly, we preincubated isolated aortic rings with either bradykinin or L-NAME or ODQ or zaprinast to find the putative involvement of the NO/cGMP-dependent signaling pathway for the inhibitory effect of VACE following PE-induced aorta muscle contractions. Specifically, our findings showed that treatment with NOS stimulator bradykinin and PDE5 inhibitor zaprinast further increased the ASM relaxant effects of VACE. Indeed, relaxation to VACE was significantly decreased by the presence of a NOS inhibitor L-NAME and an inhibitor of sGC ODQ. These results suggested the involvement of the NO-sGC-cGMP pathway in the vasorelaxant effects of VACE, by stimulating NO and cGMP synthesis and/or inhibiting cGMP degradation [41]. These may also explain the relaxation mechanism of VACE more pronounced on endothelium-intact and less effective on endothelium-denuded ASM.

Prostacyclin is an endothelium-derived specific mediator and factor contributing to vasorelaxation [42], as it has been reported in circulatory failure cases in sepsis. An important endothelium-dependent pathway participating in vascular relaxation is the PG-cAMP pathway [28, 29]. Production of PG in the endothelium is catalyzed by COX enzyme isoforms [30]. To determine whether the PG pathway is involved in VACE-induced ASM relaxation, we tested the role of indomethacin, COX-1, and COX-2 inhibitor and blocker of the PG-cAMP pathway. Indomethacin blocks PG synthase and the PG-cAMP pathway. As we have shown, pretreatment with indomethacin (Figure 4) significantly inhibited the relaxant effect of VACE in aortic rings precontracted with PE. Thus, suggesting that the aorta relaxation effect induced by VACE might be partly realized through prostaglandins' formation.

## 5. Conclusions

This study finding has proven that VACE exerts vascular relaxant properties that are endothelium-dependent and, at least partly, are mediated by NO/cGMP- and COX-1-PG-dependent mechanisms. Also, VACE may act through directly relaxing the ASM cells, by inhibiting cGMP, hydrolyzing PDEs, and other endothelium-independent mechanisms involved in vascular relaxation. Taken together, our findings might give reasons to explain the effects of VACE that seem to have a potential therapeutic value in the treatment of cardiovascular diseases, including hypertensive heart disease.

## Data Availability

The data results used to support the findings of this study are included within the article, while the original database is deposited in our laboratory (Fac. of Medicine, Uni. Prishtina).
